# Conditional Survival After Resection for Pancreatic Cancer: A Population-Based Study and Prediction Model

**DOI:** 10.1245/s10434-020-08235-w

**Published:** 2020-02-12

**Authors:** Anouk E. J. Latenstein, Stijn van Roessel, Lydia G. M. van der Geest, Bert A. Bonsing, Cornelis H. C. Dejong, Bas Groot Koerkamp, Ignace H. J. T. de Hingh, Marjolein Y. V. Homs, Joost M. Klaase, Valery Lemmens, I. Quintus Molenaar, Ewout W. Steyerberg, Martijn W. J. Stommel, Olivier R. Busch, Casper H. J. van Eijck, Hanneke W. M. van Laarhoven, Johanna W. Wilmink, Marc G. Besselink, for the Dutch Pancreatic Cancer Group

**Affiliations:** 1 Department of Surgery, Cancer Center Amsterdam, Amsterdam UMC, University of Amsterdam, Amsterdam, The Netherlands; 2 Department of Research and Development, Netherlands Comprehensive Cancer Organisation (IKNL), Utrecht, The Netherlands; 3 Department of Surgery, Leiden University Medical Center, Leiden, The Netherlands; 4 Department of Surgery, Maastricht University Medical Centre and NUTRIM School of Nutrition and Translational Research in Metabolism, Maastricht, The Netherlands; 5 Department of Surgery, Erasmus MC Cancer Institute, Rotterdam, The Netherlands; 6 Department of Surgery, Catharina Hospital, Eindhoven, The Netherlands; 7 Department of Medical Oncology, Erasmus MC Cancer Institute, Rotterdam, The Netherlands; 8 Department of Surgery, University Medical Center Groningen, Groningen, The Netherlands; 9 Department of Public Health, Erasmus Medical Center, Rotterdam, The Netherlands; 10 Department of Surgery, Regional Academic Cancer Center Utrecht, St Antonius Hospital Nieuwegein and University Medical Center Utrecht Cancer Center, Utrecht, The Netherlands; 11 Department of Public Health, Erasmus MC, Rotterdam, The Netherlands; 12 Department of Surgery, Radboud University Medical Center, Nijmegen, The Netherlands; 13 Department of Medical Oncology, Cancer Center Amsterdam, Amsterdam UMC, University of Amsterdam, Amsterdam, The Netherlands

**Keywords:** Pancreatic cancer, Conditional survival, Survival, Prediction model

## Abstract

**Background:**

Conditional survival is the survival probability after already surviving a predefined time period. This may be informative during follow-up, especially when adjusted for tumor characteristics. Such prediction models for patients with resected pancreatic cancer are lacking and therefore conditional survival was assessed and a nomogram predicting 5-year survival at a predefined period after resection of pancreatic cancer was developed.

**Methods:**

This population-based study included patients with resected pancreatic ductal adenocarcinoma from the Netherlands Cancer Registry (2005–2016). Conditional survival was calculated as the median, and the probability of surviving up to 8 years in patients who already survived 0–5 years after resection was calculated using the Kaplan–Meier method. A prediction model was constructed.

**Results:**

Overall, 3082 patients were included, with a median age of 67 years. Median overall survival was 18 months (95% confidence interval 17–18 months), with a 5-year survival of 15%. The 1-year conditional survival (i.e. probability of surviving the next year) increased from 55 to 74 to 86% at 1, 3, and 5 years after surgery, respectively, while the median overall survival increased from 15 to 40 to 64 months at 1, 3, and 5 years after surgery, respectively. The prediction model demonstrated that the probability of achieving 5-year survival at 1 year after surgery varied from 1 to 58% depending on patient and tumor characteristics.

**Conclusions:**

This population-based study showed that 1-year conditional survival was 55% 1 year after resection and 74% 3 years after resection in patients with pancreatic cancer. The prediction model is available via www.pancreascalculator.com to inform patients and caregivers.

**Electronic supplementary material:**

The online version of this article (10.1245/s10434-020-08235-w) contains supplementary material, which is available to authorized users.

Pancreatic ductal adenocarcinoma (hereafter called pancreatic cancer) is one of the most lethal cancers. In Europe and the US, approximately 18 per 100,000 persons and 13 per 100,000 persons, respectively, are diagnosed with this disease annually.^1^^,^^2^ Approximately 16% of all patients will undergo surgical resection, with a 5-year survival rate of 15–20%.^3^–^5^ Survival following resection of pancreatic cancer has improved because of better adjuvant treatment strategies.^6^^,^^7^ Therefore, increasing numbers of patients with pancreatic cancer will survive the first year following surgery and these patients might want to be informed about accurate data on survival estimates during follow-up.

Survival estimates are traditionally calculated from the time of diagnosis or from the time of surgery. However, in patients who underwent pancreatic resection for pancreatic cancer, predicted survival changes considerably during follow-up.^8^–^10^ Conditional survival (CS), defined as the survival probability and calculated in the subgroup of patients who have survived a predefined period, may therefore provide better insight. This could for instance be relevant when patients in follow-up after resection of pancreatic cancer are faced with important decisions regarding work and personal life, with impact on both themselves and their next of kin. CS may also facilitate appropriate risk stratification of patients, e.g. regarding the frequency and timing of follow-up.^8^–^13^ For optimal risk stratification, calculation of the CS probability should also take other predictors of overall survival into account. Multiple prediction models have been developed for survival after surgery for pancreatic cancer;^14^^,^^15^ however, prediction models for CS in pancreatic cancer are lacking.

The Netherlands Cancer Registry (NCR) contains patient, tumor, and treatment characteristics of all patients with pancreatic cancer, as well as corresponding survival data. The objective of this study was to assess CS using nationwide NCR data for patients who underwent resection of pancreatic cancer and to develop a nomogram to predict CS probabilities, with the possibility of adjusting survival estimates for a certain period already survived after surgery.

## Methods

### Study Design

This cohort study used nationwide data from the NCR, a prospective population-based database that covers all Dutch hospitals (i.e. a population of 16.8 million). Information on patient, tumor, and treatment characteristics from patients with a newly diagnosed malignancy are routinely collected from medical records by trained NCR administrators. Patients were queried from the national pathological archive (PALGA) and the National Registry of Hospital Discharge Diagnoses. This study was reported in accordance with the STROBE guidelines.^16^ No informed consent was required as anonymized data were used.

### Study Population

Patients who underwent resection of pancreatic ductal adenocarcinoma during the period 2005–2016 were extracted from the NCR database (International Classification of Diseases for Oncology, Third Revision [ICD-O-3] morphology codes are shown in electronic supplementary Text 1). Pancreatic resection was defined as pancreatoduodenectomy, distal pancreatectomy, or total pancreatectomy. Patients younger than 18 years of age at the time of diagnosis, patients with neuroendocrine tumors, and patients with distant metastases were excluded. In addition, patients, who died within 30 days after surgery were excluded since our aim was to develop a nomogram for postoperative use in the outpatient clinic.

### Data Collection

Primary tumor location was classified as the pancreatic head, body, tail, or other/not otherwise specified (NOS), according to the ICD-O-3. Staging was based on pathological classification according to the TNM classification at the time of registration (6th edition of the Union for International Cancer Control [UICC] TNM staging during 2005–2009; 7th edition of the UICC TNM staging during 2010–2016).^17^^,^^18^ In case of neoadjuvant treatment or missing pathological TNM stage, the clinical TNM classification was used. Adjuvant chemotherapy has been recommended since 2008 after judgment of a national commission (Commissie BOM), and was, according to the guidelines, almost universally gemcitabine only. Survival data were obtained by an annual cross-check with the Municipal Personal Records Database, which contains the vital status of all Dutch inhabitants. Survival was calculated as the time between the date of surgery (or date of histological diagnosis when the date of surgery was unknown, *n* = 3) and date of death, or censored when alive at the last check of the patient’s vital status (1 February 2018).

### Statistical Analysis

Patient, tumor, and treatment characteristics were presented using descriptive statistics. Overall survival was calculated using the Kaplan–Meier method. CS was defined as the probability of surviving an additional number of ‘*y*’ years, given that a patient had already survived for ‘*x*’ years, and was calculated as CS_(*x*|*y*)_ = *S*_(*x*+*y*)_/*S*_(*x*)_, with *S*_(*x*)_ representing the overall survival at *x* years estimated using the Kaplan–Meier method.^12^ For example, to estimate the CS for surviving 2 more years for patients who had already have survived 3 years after surgery, CS_(3|2)_ is calculated by dividing the 5-year Kaplan–Meier survival estimate *S*_(5)_ by the 3-year Kaplan–Meier survival estimate *S*_(3)_.^8^^,^^19^–^21^ Median CS was also determined at specific times and was derived from Kaplan–Meier estimates by discarding the patients who died before that time.

To develop a nomogram predicting 5-year survival, the predictors of the previously published and externally validated Amsterdam model were used.^14^^,^^22^ This model was used because of its simplicity and methodological quality according to a recent systematic review and to maintain consistency with previous studies.^14^^,^^23^ The Amsterdam model uses adjuvant chemotherapy, margin status, tumor differentiation, and lymph node ratio to predict overall survival for patients who underwent pancreatoduodenectomy for pancreatic cancer.^14^ Moreover, age was also incorporated in the prediction model because of its relation with CS. In the current study, multiple imputation was used to impute missing data by creating 10 datasets, using the *mice* package in R. Variables of the Amsterdam model were included in a multivariable Cox proportional hazards model. A penalized LASSO model (Least Absolute Shrinkage and Selector Operator) was used in order to enhance prediction accuracy and reduce overfitting.^24^ Results were presented as hazard ratios (HR) with 95% confidence intervals (CIs). A nomogram was created and the C-statistic was presented, with optimism adjusted for by bootstrapping (*B* = 200). Nomogram-predicted CS rates to reach 5-year survival were presented directly after surgery and given 1, 2, 3, and 4 years survival after surgery (for use in the outpatient clinic during follow-up). Of note, CS predictions ‘directly after surgery’ are actually the predictions at 30 days post-surgery (since 30-day mortality was excluded), but was described as ‘directly after surgery’ to enhance readability. All *p* values were based on a two-sided test and *p* values < 0.05 were considered statistically significant. Statistical analysis was performed using IBM SPSS Statistics for Windows version 25 (IBM Corporation, Armonk, NY, USA) and R version 3.4.3 (cran.r-project.org).

## Results

In total, 3204 patients underwent resection of pancreatic cancer between 2005 and 2016. Patients who died within 30 days after surgery were excluded (4%, *n* = 122). The final cohort consisted of 3082 patients; median age was 67 years (interquartile range 60–73) and 1630 patients (53%) were male. All baseline characteristics are shown in Table 1.

**Table 1:** Baseline characteristics of 3082 patients with resected pancreatic cancer diagnosed between 2005 and 2016

Clinicopathological parameters	Total cohort^a^ [*n* = 3082]
Male	1630 (53)
Age, years [median (IQR)]	67 (60–73)
< 70	1892 (61)
≥ 70	1190 (39)
Primary tumor location	
Head of the pancreas	2509 (81)
Corpus of the pancreas	110 (3.6)
Tail of the pancreas	235 (7.6)
Pancreas, NOS	228 (7.4)
Type of operation	
Pancreatoduodenectomy	2686 (87)
Distal pancreatectomy	333 (11)
Total pancreatectomy	47 (1.5)
Other/NOS	16 (0.5)
Tumor differentiation grade	
Well-differentiated (grade I)	360 (12)
Moderately differentiated (grade II)	1626 (53)
Poorly or undifferentiated (grade III)	1096 (36)
Missing	484 (16)
Pathological T stage^b^	
T1	222 (7.2)
T2	555 (18)
T3	2167 (70)
T4	138 (4.5)
Pathological N stage^c^	
N0	1000 (32)
N1	2082 (68)
Resection margin status	
R0	2065 (67)
R1	966 (31)
R2	51 (1.6)
Missing	132 (4.3)
Neoadjuvant chemo(radio)therapy	140 (4.5)
Adjuvant chemotherapy	1492 (48)

Data are expressed as *n* (%) unless otherwise stated

*IQR* interquartile range, *NOS* not otherwise specified

^a^Imputed data are presented. Percentages are separately calculated for the group of missing values, explaining the cumulative exceeding 100% for tumor grade and resection margin status

^b^Clinical T stage was used in case of missing pathological T stage (*n* = 26, 0.8%)

^c^Clinical N stage was used in case of missing pathological N stage (*n* = 49, 1.6%)

### Overall and Conditional Survival

**Fig. 1:**
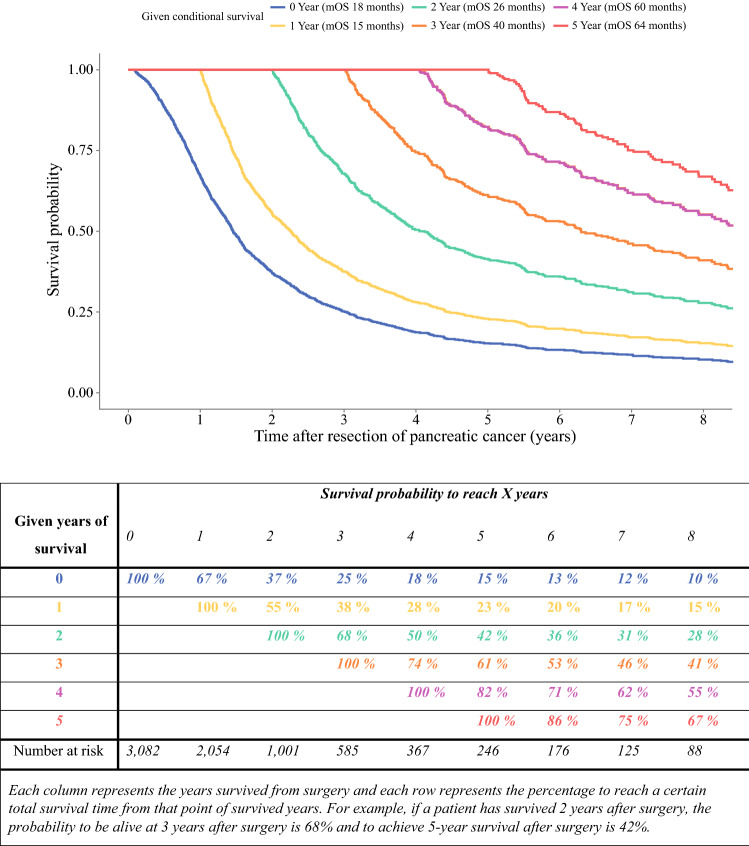
Kaplan–Meier estimates for conditional survival up to 8 years in 3082 patients given 0–5 years’ survival after resection of pancreatic cancer

Median overall survival was 18 months (95% CI 17–18 months), with a 5-year survival of 15% (Fig. 1). The survival probability increased per year already survived relative to the total survival time. The probability of achieving 5-year survival after resection increased from 15% directly after surgery to 23%, 42%, 61%, and 82% per additional year survived (i.e. 1, 2, 3, and 4 years after resection, respectively). The 1-year CS (i.e. probability of surviving the next year) decreased from 67% directly after surgery to 55% at 1 year after surgery, and then increased to 74% and 86% at 3 and 5 years after surgery, respectively (Fig. 1). The median CS decreased from 18 months (95% CI 17–18) directly after surgery to 15 months (95% CI 14–16) at 1 year after surgery, and then increased to 40 (95% CI 32–52) and 64 months (95% CI 54—not reached) at 3 and 5 years after surgery, respectively.

### Multivariable Analysis of Survival

In our cohort, all four variables of the Amsterdam model (i.e. adjuvant chemotherapy, margin status, tumor differentiation, and lymph node ratio), as well as age, were independent predictors of survival in a multivariable Cox analysis (Table 2). Moderately and poorly differentiated tumors were associated with worse survival compared with well-differentiated tumors (HR 1.27 [95% CI 1.11–1.46] for moderately differentiated tumors, and HR 1.74 [95% CI 1.51–2.00] for poorly/undifferentiated tumors). In addition, higher lymph node ratio and an R1/R2 resection margin were independently associated with decreased survival, as was the absence of use of adjuvant chemotherapy (HR 1.64 [95% CI 1.51–1.79]).

**Table 2:** Univariable and multivariable Cox regression analyses according to the Amsterdam model in patients with resected pancreatic cancer diagnosed between 2005 and 2016

Clinicopathological parameter	Median OS, months	5-year survival (%)	Univariable analysis HR (95% CI)	Multivariable analysis HR (95% CI)^b^	*p* value^c^
Age (each incremental year)	–	–	1.01 (1.00–1.01)	1.00 (1.00–1.01)	0.04
Tumor differentiation grade					
Well-differentiated	27	27	1.00 (reference)	1.00 (reference)	
Moderately differentiated	19	16	1.41 (1.21–1.65)	1.27 (1.11–1.46)	0.001
Poorly or undifferentiated	14	12	1.94 (1.66–2.28)	1.74 (1.51–2.00)	< 0.001
Lymph node ratio^a^					
0 (lymph node-negative)	25	28	1.00 reference	1.00 (reference)	
> 0 and ≤ 0.18	18	13	1.44 (1.27–1.63)	1.47 (1.31–1.64)	< 0.001
> 0.18	15	8	1.86 (1.67–2.07)	1.94 (1.76–2.14)	< 0.001
Resection margin					
R0	20	19	1.00 (reference)	1.00 (reference)	
R1/R2	14	8	1.57 (1.44–1.70)	1.44 (1.33–1.57)	< 0.001
Adjuvant chemotherapy					
Yes	21	20	1.00 (reference)	1.00 (reference)	
No	14	11	1.52 (1.41–1.65)	1.64 (1.51–1.79)	< 0.001

Data after multiple imputation were used

*OS* overall survival, *HR* hazard ratio, *CI* confidence interval

^a^Lymph node ratio is the number of positive lymph nodes divided by the total number of lymph nodes harvested

^b^Hazard ratios and 95% CIs from the Cox LASSO model are presented

^c^*p* values of multivariable analyses are shown

### Prediction Nomogram for Conditional Survival

**Fig. 2:**
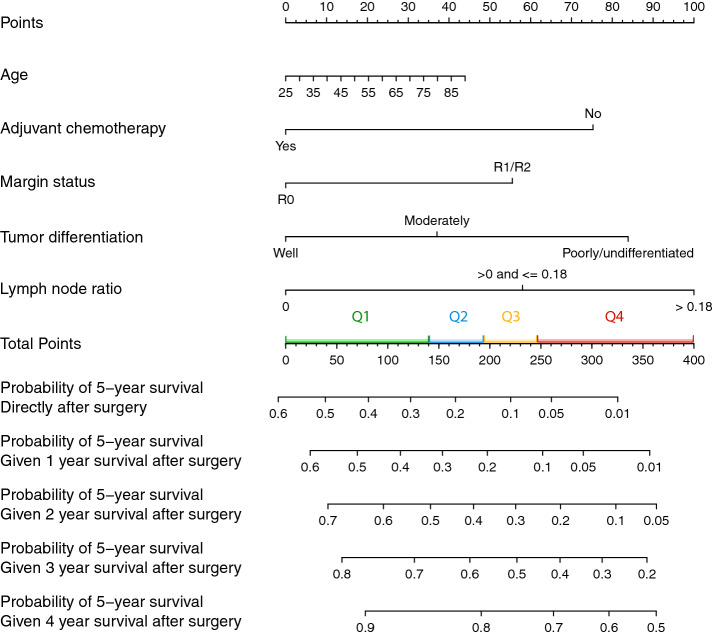
Nomogram for the prediction of overall and conditional survival to achieve 5-year survival after resection. For a given patient per variable of the nomogram, locate the corresponding value and draw a vertical line upward toward the ‘Points’ axis. Add the points for all four variables and draw a vertical line from this number of points from the ‘Total Points’ axis downwards through the probability axes. This will indicate the patient’s probability to reach 5-year survival directly after surgery and 1, 2, 3, and 4 years after surgery

In Fig. 2, a nomogram was created based on the predictors of the multivariable Cox model. The prediction model had a calibration slope of 1.1 (electronic supplementary Fig. 1) and an optimism-adjusted C-statistic of 0.65 (95% CI 0.64–0.66). The nomogram predicts the probability of reaching 5-year survival directly after surgery and after surviving 1–4 years after surgery. Quartiles of the nomogram score are indicated in the nomogram to show the distribution of the current cohort. The probability of achieving 5-year survival, measured 1 year after surgery, varied from 1 to 58% depending on patient and tumor characteristics. For example, a 60-year-old patient with a moderately differentiated tumor and a lymph node ratio of < 0.18 who underwent an R1 resection without adjuvant chemotherapy would have a total nomogram score of 249 (24 + 37 + 58 + 55 + 75). The probability of being alive 5 years after surgery was 10% after surviving the first year for this particular patient, CS_(5|1)_, increasing to 45% when surviving the first 3 years after surgery, CS_(5|3)_. If this patient had received adjuvant chemotherapy, the total nomogram score would have been 174 points (24 + 37 + 58 + 55) and the probability of 5-year survival would have been 25% after surviving the first year, rising to 61% after surviving the first 3 years after surgery, CS_(5|3)_.

A calculator to estimate the probability of achieving 5-year survival, calculated from the time of surgery, given ‘*x*’ years of survival after surgery, has been made available at www.pancreascalculator.com.

## Discussion

This nationwide study in 3082 patients who underwent resection of pancreatic cancer is the first to present a prediction model for CS. The probability of achieving 5-year survival after pancreatic resection increased from 15% directly after surgery to 61% after surviving the first 3 years. A prediction model was created, using easily accessible predictors, and has been made available at www.pancreascalculator.com to estimate patient-specific CS probabilities for 5-year survival after surgery.

CS is especially of interest in cancers with a poor survival prognosis as the survival estimates change considerably after surviving the first year. In the current study, the 1-year CS (i.e. the probability of surviving another year) decreased the first year after surgery (67% directly after surgery vs. 55% at 1 year after surgery). This indicates that relatively more patients die in the second year after surgery than in the first year after surgery. After this initial decrease, the 1-year CS estimates gradually increase. The large decline in survival in the second year after surgery is merely a reflection of the non-linear death rate in patients diagnosed with pancreatic cancer. In other large series, disease recurrence also typically occurs after a median of 12 months.^25^ Patients who have survived the first years after surgery probably have less aggressive cancers. This is also confirmed by the different shapes of the CS curves in Fig. 1 (the concave becoming more linear over time). Another explanation might be extensive patient care with optimization of the physical condition perioperatively and during the first year postoperatively. After the first year, oncological treatments are typically completed and the intensity of supportive care potentially decreases. However, the exact reason of the biggest decline in the second year after surgery remains unknown.

The increase in the CS after these first years is probably because only patients who had a tumor with favorable biological behavior remain. These patients survive until late tumor recurrence or other causes of death, leading to an increased CS as patients have accrued a longer postoperative survival. Moreover, distinction between pancreatic, ampullary and distal bile duct cancer remains challenging, while these cancers carry different prognoses.^26^ Tumors might be misclassified as pancreatic ductal adenocarcinoma and patients could therefore have a better survival than expected, being translated in increasing CS over time.

The survival in this study is lower compared with other large, monocenter series;^27^^,^^28^ however, this is a population-based study and the results are therefore more representative than studies with selected cohorts, for example from single, high-volume centers. Compared with the population-based Surveillance, Epidemiology, and End Results (SEER) database, our results are similar.^29^

The current CS estimates are developed for the outpatient clinic after full recovery from surgery and when patients would like to discuss their prognosis and future perspectives. Our nomogram uses readily available and widely recognized predictors of survival in pancreatic cancer. Although some might argue that a C-statistic of 0.65 is relatively low, it is in line with previous prediction models in pancreatic cancer.^23^ The difficulty in accurately predicting survival after resected pancreatic cancer is partly related to the narrow-banded survival distribution (poor prognosis for the vast majority of patients with very few long-term survivors), which complicates accurate discrimination in terms of clinical outcome.

Recently, other studies reported on CS in colorectal liver metastases, hepatocellular carcinoma, non-small lung cancer, and malignant brain tumors.^20^^,^^21^^,^^30^^,^^31^ However, in pancreatic cancer, only a few, mostly single-center studies have assessed CS without taking other prognostic factors into account.^8^–^11^^,^^13^^,^^32^^,^^33^ One European study analyzed CS among all stages of pancreatic cancer, stratified for age and sex, but presented only limited information on CS.^32^ Another recent study combined data from Verona and Boston and stratified for TNM stage, tumor grade, resection margin, and adjuvant therapy.^33^ This study separated patients with and without tumor recurrence. Unfortunately, this was not possible in our cohort since this information was not yet available in the NCR during 2005–2016. Comparison of the overall population analysis from that study with our results showed that 1-year CS was slightly higher in their study, but this effect diminished over time.^33^ Moreover, a recent study including five national cancer registries developed a survival-predicting model for 1-, 2-, 3-, and 5-year survival probabilities.^34^ CS was not calculated in this large cohort. However, none of the previously mentioned series proposed a way to calculate CS with adjustment for known clinicopathological predictors. As known from previous studies, not only time since resection affected overall survival but obviously also patient, tumor, and treatment characteristics.^27^^,^^35^^,^^36^ In studies on CS for gastric cancer, a nomogram to adjust for covariates was created and consequently increased the accuracy of CS estimates.^37^^,^^38^

Patients might be unable to adequately interpret traditional 3- and 5-year survival estimates, potentially leading to rigorous decisions. The nomogram created in the current study will potentially add to traditional survival estimates in counselling patients and surveillance during follow-up. Moreover, patients prefer explicit information about prognosis.^39^ Some patients might experience anxiety as 3 years after surgery is approaching, while this study demonstrates that CS rates are actually improving over time. These psychological consequences, such as fear of cancer recurrence or death, become more important due to novel and improved treatment possibilities that increase survival.^40^ Personalized survival estimates will potentially aid to deal with these psychological factors and will pave the way for personalized follow-up schedules. Furthermore, as can be calculated with the prediction model, patients with adjuvant chemotherapy have higher CS estimates compared with patients without chemotherapy. These estimates might increase the patients’ visualization of the impact of adjuvant chemotherapy on survival. Based on these estimates, one might also cautiously advocate for the treatment of oligometastatic disease after 2–3 years progression-free survival as CS probabilities are improving over time.

This study has some limitations. First, the retrospective design could have caused bias because surgical and pathological procedures were not standardized among centers. For example, the pathological assessment of pancreatic resection specimens improved considerably during these years (2005–2016), which we were not able to adjust for retrospectively and might have influenced our results. Second, one of the strengths of this study, the long study period, also represents one of its limitations. Surgical outcomes improved due to increased centralization, and new (neo)adjuvant chemotherapy regimens were introduced.^6^, ^41^–^44^ It is likely that the majority of patients received adjuvant gemcitabine monotherapy, whereas now most patients receive (neo)adjuvant FOLFIRINOX, resulting in an improved survival.^6^ Unfortunately, in our cohort, only a small proportion of patients was treated neoadjuvantly as this was only done in randomized trials during these years. With new insights available and treatment shifting rapidly towards neoadjuvant therapy, the current CS estimates are probably an underestimation of the actual prognosis. An update of the nomogram would be appropriate in a few years due to these improvements, perhaps including the type of chemotherapy and completeness of chemotherapy regimens. Third, no data were available on tumor recurrence and cancer antigen (CA) 19-9. Recurrence has a considerable prognostic impact, as was shown in the study from Verona and Boston.^33^ The NCR database is currently expanded with recurrence data, and, subsequently, further research should incorporate these data to improve patient-tailored calculations. CA19-9 is a tumor marker that was shown to be of prognostic value but was not yet registered in a considerable proportion of the patients included in our cohort and could therefore not be considered in our analysis.^26^ Fourth, it should be noted that the number of patients at risk in the CS analysis substantially decreased over time. Smaller groups obviously result in wider CIs, especially longer after surgery, which should be taken into account. Due to the statistical challenges to calculate CIs of the CS Kaplan–Meier estimates, the number of patients at risk is presented instead.

## Conclusion

This nationwide study describes CS following resection of pancreatic cancer. A nomogram and online calculator based on national data may be useful for counselling patients during follow-up. External validation of the nomogram and CS estimates in other cohorts of patients with pancreatic cancer would be recommended.

## Electronic supplementary material

Below is the link to the electronic supplementary material.

Supplementary material 1 (DOCX 136 kb)
