# Supplementary material for: Conditional Survival After Resection for Pancreatic Cancer: A Population-Based Study and Prediction Model
Source: Ann Surg Oncol. 2020 Feb 12;27(7):2516–24. doi: 10.1245/s10434-020-08235-w (PMC7311496; doi:10.1245/s10434-020-08235-w)

**Supplementary files**

**Supplementary Text 1**

Patients were included based on the ICD-O-3 morphology codes below. One patient, who underwent pancreatic resection was coded with code 8010. This patient was also included.

8012 Large cell carcinoma, NOS

8020 Carcinoma, undifferentiated, NOS

8021 Carcinoma, anaplastic, NOS

8022 Pleomorphic carcinoma

8031 Giant cell carcinoma

8032 Spindle cell carcinoma, NOS

8033 Pseudosarcomatous carcinoma

8035 Carcinoma with osteoclast-like giant cells

8046 Non-small cell carcinoma

8070 Squamous cell carcinoma, NOS

8071 Squamous cell carcinoma, keratinizing, NOS

8072 Squamous cell carcinoma, large cell, nonkeratinizing, NOS

8140 Adenocarcinoma, NOS

8141 Scirrhous adenocarcinoma

8143 Superficial spreading adenocarcinoma

8144 Adenocarinoma, intestinal type

8145 Carcinoma, diffuse type

8163 Pancreatobiliary-type carcinoma

8201 Cribiform carcinoma, NOS

8211 Tubular adenocarcinoma

8255 Adenocarcinoma with mixed subtypes

8310 Clear cell adenocarcinoma, NOS

8480 Mucinous adenocarcinoma

8481 Mucin-producing adenocarcinoma

8490 Signet ring cell carcinoma

8500 Ductal carcinoma, NOS

8510 Medullary carcinoma, NOS

8521 Infiltrating ductular carcinoma

8523 Infiltrating duct mixed with other types of carcinoma

8560 Adenosquamous carcinoma

8570 Adenocarcinoma with squamous metaplasia

8572 Adenocarcinoma with spindle cell metaplasia

8575 Metaplastic carcinoma

8576 Hepatoid adenocarcinoma

**Figure 1. The calibration plot for prediction of 5-year survival**

**Figure 2. Example of** [**www.pancreascalculator.com**](http://www.pancreascalculator.com) **based on a 60-year old patient with a moderately differentiated tumor and a lymph node ratio of <0.18 who underwent an R1 resection with adjuvant chemotherapy.**


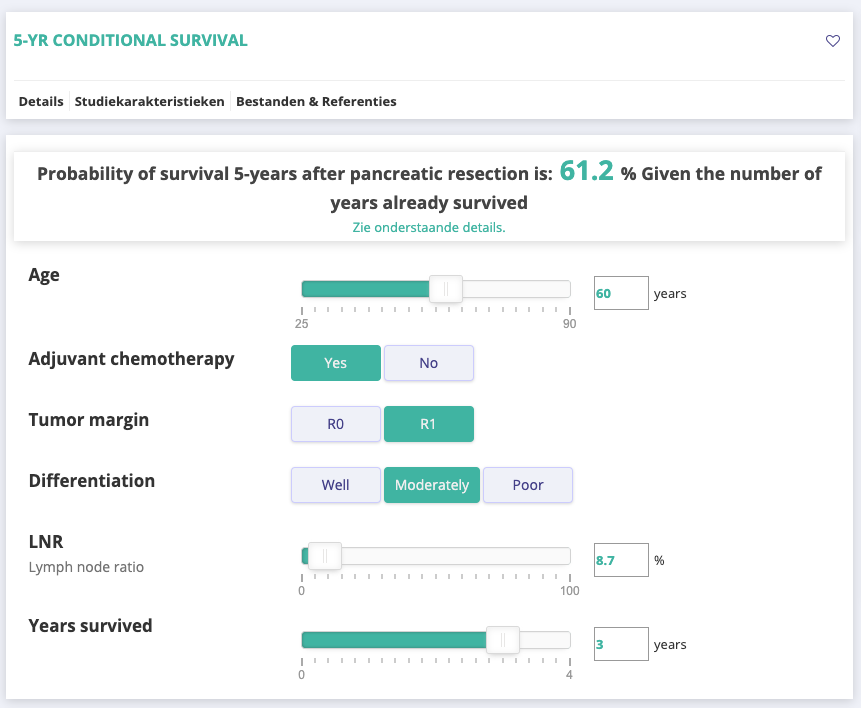

Supplement: Supplementary file 1 — Supplementary material 1 (DOCX 136 kb) [file 10434_2020_8235_MOESM1_ESM.docx]
